# Recombinant Ranaviruses for Studying Evolution of Host–Pathogen Interactions in Ectothermic Vertebrates

**DOI:** 10.3390/v8070187

**Published:** 2016-07-06

**Authors:** Jacques Robert, James K. Jancovich

**Affiliations:** 1 Department of Microbiology and Immunology, University of Rochester Medical Center, Rochester, NY 14642, USA; 2 Department of Biological Sciences, California State University San Marcos, 333 S. Twin Oaks Valley Rd., San Marcos, CA 92096, USA; jjancovich@csusm.edu

**Keywords:** Amphibians, Xenopus, iridovirus, DNA viruses, reporter virus, recombinant virus

## Abstract

Ranaviruses (*Iridoviridae*) are large DNA viruses that are causing emerging infectious diseases at an alarming rate in both wild and captive cold blood vertebrate species all over the world. Although the general biology of these viruses that presents some similarities with poxvirus is characterized, many aspects of their replication cycles, host cell interactions and evolution still remain largely unclear, especially in vivo. Over several years, strategies to generate site-specific ranavirus recombinant, either expressing fluorescent reporter genes or deficient for particular viral genes, have been developed. We review here these strategies, the main ranavirus recombinants characterized and their usefulness for in vitro and in vivo studies.

## 1. Introduction

Ranavirus pathogens are causing emerging infectious diseases in many ectothermic vertebrate species worldwide [1,2]. The increase in prevalence and in the range of infected hosts from fish and amphibians to reptiles is alarming for biodiversity in the wild as well as for aquaculture and international trade of animals [3,4,5]. Importantly, ranaviruses are capable of crossing species barriers of numerous ectothermic vertebrates, suggesting that these pathogens possess potent immune evasion strategies [6,7,8]. Furthermore, although some species are highly susceptible to ranaviruses, others are more resistant and can serve as asymptomatic carriers for viral dissemination [9,10].

The World Organization for Animal Health (OIE) lists ranaviruses that infect amphibians as notifiable pathogens, which means that international trade of live amphibians and related products should require health certifications to be applied according to OIE standards. In addition, recent warnings about the international trade of amphibians in the dissemination of this disease highlight the importance of these pathogens [11]. However, movement of ranavirus host species continues and with that the increased probability of further pathogen dissemination. As ranaviruses continue to influence wild and cultured cold-blooded vertebrate populations, it is crucial to better understand the molecular biology driving these infectious agents.

Ranaviruses are large, icosahedral, double-stranded DNA viruses that belong to the family *Iridoviridae*, which is part of the monophyletic group of nucleocytoplasmic large DNA viruses (NCLDV) that also includes *Poxviridae* [12]. Ranaviruses possess large genomes, ranging between 105 and 155 kilobase pairs in size and encoding 95 to 162 open reading frames (ORFs; [12]). Despite their growing economic and ecological significance, ranaviruses have not been as extensively studied as other families of large double-stranded DNA (dsDNA) viruses (e.g., *Poxviridae* and *Herpesviridae*), and their mechanisms of replication, infection, and pathogenesis are still poorly understood.

Based on early studies using *Frog virus* 3 (FV3), which is the type species of the genus [13], and more recent work with other ranaviruses, the general outlines of the ranavirus replication cycle are known. Similar to poxvirus, no cellular receptor critical for viral entry has been identified to date for these promiscuous viruses that can infect many different cell types in vitro and in vivo [14]. In fact, ranaviruses can multiply at 32 °C or lower even in mammalian and avian cells, although they cannot replicate at 37 °C [15].

A distinctive feature of FV3 and other ranaviruses is that a viral envelope added onto virions budding from the plasma membrane of infected cells is not required for infectivity. As such naked virus particles released during cell lysis are infectious. However, naked viral DNA is not infectious, which suggests that virion-associated proteins are required for infection. Viral entry into the cytoplasm is thought to occur by receptor-mediated endocytosis for enveloped virions or by release of viral DNA into the cytoplasm by naked virus [12,16,17].

In contrast to poxviruses that replicate only in the cytoplasm, ranaviruses present the peculiarity of replicating both in the nucleus and in the cytoplasm of infected cells. After viral entry, early viral transcription and a first round of unit length genome replication take place within the nucleus using the host DNA polymerase. At later stages, when the viral DNA polymerase is produced, genomes are transported into the cytoplasm and serve as templates for concatemer formation. Ranavirus genomic DNA is both circularly permuted and terminally redundant [18], leading to a genome map that is circular, whereas the actual molecule is still linear [19]. In addition, ranavirus genomes are highly methylated, a feature unique among animal viruses. This is another marked difference, particularly among genera within the family *Iridoviridae*, as ranavirus genomes are highly modified with greater than 20% of its cytosine CpG sequences methylated [20]. While the function of viral methylation has not been conclusively determined, the high level of methylation suggests an important evolutionary advantage for this group of viruses.

Ranavirus virions are assembled in cytoplasmic assembly sites that contain viral DNA and various virus-encoded proteins. Newly formed ranavirus particles can be found free within the cytoplasm, accumulated in para-crystalline arrays or bud from the plasma membrane and acquire an envelope. Recently, work with Singapore grouper iridovirus, a ranavirus related to FV3, suggests that ranavirus maturation and envelope formation can also occur intracellularly using cytoplasmic vesicles [21]. Mature naked and enveloped virions released help spread the viral infection locally and into the environment. In many host species, ranaviruses can infect almost every tissue type in a host and median lethal dose (LD_50_) studies provide supportive evidence of the infectious power of ranavirus pathogens [22].

To date, as many as 19 iridovirus genomes have been sequenced [1,12,23,24,25]. However, precise functions of most ranaviral genes are still unknown. For example, only about one-third of FV3’s ~100 genes have been assigned putative functions and this mostly by sequence homology to known viral and host genes. As a result, there is a limited knowledge of the host–pathogen interactions between ranaviruses and their cold-blooded vertebrate hosts. Because of the increasing role of ranaviruses in amphibian declines and disease outbreaks among commercially important amphibian and fish species, it is imperative to gain better insight into the determinants of virulence encoded by these emerging pathogens as well as to be able to trace viral infection in vivo.

Here, we review recent progress in generating recombinant ranaviruses with a special emphasis on the characterization of putative virulence or immune evasion genes and using expression of reporter fluorescent gene under viral promoter to trace viral infection.

## 2. *Ambystoma Tigrinum Virus* (ATV)

The *Ambystoma Tigrinum Virus* (ATV) was first isolated from tiger salamanders in southern Arizona during an epizootic in the San Rafael Valley in 1995 and the genome sequenced [26]. Interestingly, ATV encodes around 95 ORFs, yet less than half of these ORFs have a predicted function.

The first recombinant ATV (rATV) has the viral gene for the homolog of the eukaryotic translation initiation factor 2 α (vIF2α) replaced with the selectable marker neomycin [27]. This rATV containing the *neoR* gene was generated by homologous recombination between viral DNA and a linear DNA recombination cassette. The recombination cassette was constructed by overlapping PCR; it contained 1200 nucleotides (nt) of up- and down-stream homologous sequences flanking an ATV promoter and 200 nt of sequence upstream of the immediate early *ICP-18* ATV gene fused to the *neoR* gene. rATV can be selected, isolated and purified because of the sensitivity of wild-type ATV (wtATV) to neomycin. Cells are infected with wtATV and then transfected with the linear recombination cassette. The use of this linear recombination cassette DNA molecule allows for the generation of a recombinant virus lacking the target gene, in this case the 57R ORF that is neomycin resistant. Neomycin resistance can only be propagated in progeny virions if two recombination events take place—one with the left (i.e., upstream) flanking sequence and a second with the right (i.e., downstream) flanking sequence. Subsequent rounds of selection inhibit wtATV growth, while promoting rATV growth. Therefore, recombinant virus can be enriched for, and eventually, isolated in pure culture and characterization of viral replication in the absence of the gene can be carried out both in vitro and in vivo. In ATV, the deletion of the *vIF2α* gene (ORF 57R) resulted in modulation of protein kinase PKZ, a molecule resembling protein kinase R (PKR) in fish and amphibians [28,29,30]. PKR is the interferon inducible double-stranded RNA protein kinase R that phosphorylates the eukaryotic translation initiation factor 2-α (eIF2α), thereby inhibiting protein synthesis [31,32,33]. The deletion of 57R in ATV resulted in degradation of PKZ in transiently transfected fathead minnow cells (FHM) and sensitivity to FHM interferon [27]. In addition, ATVΔ57R showed reduced pathogenesis in tiger salamanders providing support that this gene enhances viral replication and pathogenesis. Interestingly, when the ATV 57R gene was inserted into the vaccinia virus (VACV) *E3L* locus, a gene that modulates PKR activation by binding and sequestering dsRNA produced by the virus [34,35], it could not rescue the deletion of *E3L* in vivo and suggested a role in interferon sensitivity in vitro [36].

**Figure 1:**
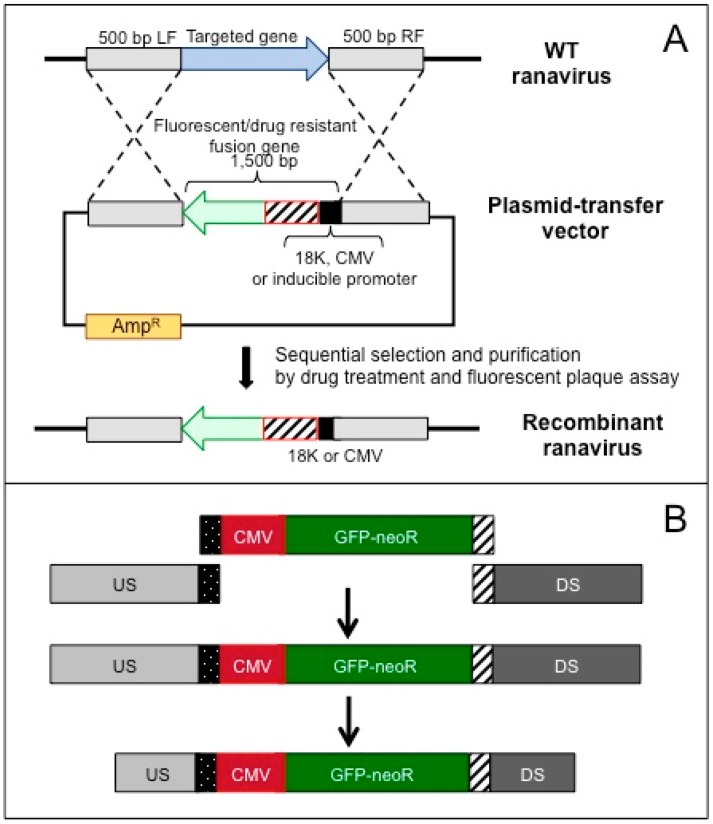
(**A**) Schematic for generating ranavirus recombinant by site-specific integration of a selection cassette. This cassette consists of a fluorescent reporter gene fused to a drug resistance gene by a short linker that is under the control of a ranaviral or ectopic promoter. This cassette is flanked by a left and right sequence portion (500 bp) of the targeted site and is cloned into a convenient bacterial plasmid. Cells are transfected with the construct using lipofectin and then infected with *wt* ranavirus to generate homologous recombination. The selection is performed sequentially by virus replication in the presence the drug and then by isolation of fluorescent plaques. (**B**) Schematic representation of the standardized process to generate a recombination cassette for *Ambystoma tigrinum virus* (ATV). Primers are designed to amplify the neomycin resistance (GNR) cassette as well as approximately 1000 nt of the upstream (US) and downstream (DS) flanking sequences for each target open reading frame (ORF). Adapter sequence added to the 3′ end of the US sequence and the 5′ end of CMV promoter. In addition, a different adapter is added to the 3′ end of the cytomegalovirus promoter (CMV-GNR) cassette and the 5′ end of the DS sequence. A standardized overlapping PCR protocol assembles the recombination cassette that is then agarose gel purified and re-amplified using primers that truncate the US and DS sequences t. This PCR product is then used to generate a recombinant virus.

Recently, a simplified, reliable and standardized process to identify essential and non-essential genes in ATV by homologous recombination has been developed [37]. This process uses a linear DNA molecule expressing a fusion protein containing green fluorescent protein (GFP) and neomycin resistance (referred to as GNR) that is expressed using a cytomegalovirus (CMV) promoter (cassette referred to as CMV-GNR) to replace ORFs of interest in the viral genome. If the target ORF is non-essential, green, neomycin resistant plaques can be isolated and purified (Figure 1A), and subsequently, if the gene is essential, a rATV cannot be easily generated. Notably, the use of an ectopic promoter diminishes the risk of recombination between the transgene and other ranaviral promoters. This approach can be used to scan for essential and non-essential genes in ATV and can easily be adapted for use in other ranavirus systems. To simplify and accelerate assembly, we have designed a PCR based amplification and cloning strategy using adaptor sequences (Figure 1B). This methodology standardizes assembly and generation of mutant virus.

## 3. *Frog virus 3* (FV3)

FV3, the main member and the type species of the genus *Ranavirus* [13], was originally isolated from the native North American leopard frog *Rana pipiens.* FV3 and FV3-like viruses are now found worldwide infecting different genera and species of amphibians, fish and reptiles, making it a potentially serious global threat to ectothermic vertebrates.

Initial attempts to identify genes essential for FV3 replication involved the isolation of a number of temperature-sensitive mutants [38,39,40]. More recent alternative ways to elucidate the function of several viral genes used transient knock down of viral gene by antisense morpholino oligonucleotides [41] or small interfering RNA (siRNA) [42]. However, the random nature of temperature sensitive mutants and the inability to readily perform knock down in vivo, have limited the usefulness of these approaches, especially if one wishes to target virulence genes that are usually non-essential for viral replication in vitro. Thus, to advance the characterization of ranaviral genes, the development of a stable, reliable and efficient site-specific mutagenesis methodology similar to that used with other DNA virus such as poxvirus, is paramount.

Although a traceable fluorescent gene reporter such as GFP is often used to screen and isolate recombinant virus ([43]; see next section), the relatively high ranavirus recombination rate could make the insertion of this gene unstable. Therefore, similar to the strategy used for ATV, a more robust, dual-selection system consisting of the puromycin-resistance gene fused to the gene for enhanced green fluorescent protein, PuroR/GFP was chosen [44]. This method is based on the use of plasmid constructs in which the PuroR/GFP gene is located downstream from the highly active 18K immediate-early promoter and the cassette flanked by several hundred nucleotides derived from sequences immediately adjacent to the genes of interest. Rather than fish or amphibian cell line, this method takes advantage of a mammalian cell line, the baby hamster kidney (BHK) cell line that supports culture at 30 °C necessary for FV3 replication. BHK cells do not generate interferon response and the higher temperature accelerates the production of high titer virus.

Typically, BHK cells are infected with wtFV3 and transfected two hours later with the PuroR/GFP plasmid. Virus harvested two days later is subjected to four rounds of selection in the presence of 50 µg/mL puromycin, followed by 4–5 rounds of selection for plaques expressing GFP (Figure 1A). This procedure has proved highly effective in isolating KO mutants. It is noteworthy that puromycin selection is also important to ensure that the cassette is not lost during large-scale production of recombinant virus.

### 3.1. GFP Knock-in FV3

**Figure 2:**
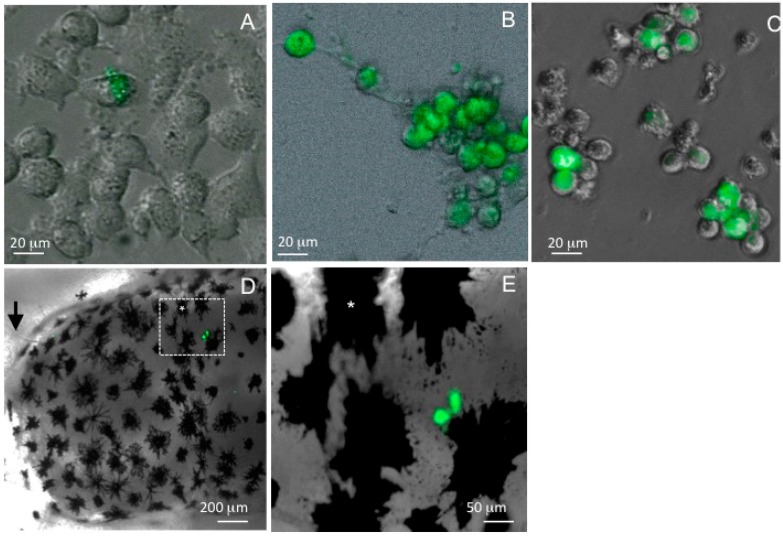
Detection of FV3-GFP knock-in mutant expressing GFP reporter under the control of the immediate early 18K promoter during infection in vitro in mammalian cell lines and in vivo in *X. laevis* tadpoles. (**A**) BHK cells at 2 h post-infection at permissive (30 °C) temperature; (**B**) mouse BV2 macrophage-like microglial cells at 24 h post-infection at permissive (30 °C) temperature; (**C**) mouse sertoli macrophage TM4 at 24 h post-infection at non-permissive (37 °C) temperature; and (**D**,**E**) midbrain view of pre-metamorphic tadpole brain at 1 day post-infection at low (**D**) and higher (**E**) magnification. (*) Indicates the same melanophore in panel D and E. Images are composite of phase contrast and fluorescence for cells (**A**–**C**) and of bright field and fluorescence of the whole-mounted tadpole, taken under a Leica DMIRB inverted fluorescence microscope and Infinity 2 digital camera (objectives ×5/×10/×20; Zeiss). Digital images were analyzed and processed by ImageJ software.

As a first FV3 recombinant, the PuroR/GFP cassette was integrated into a noncoding genomic region to serve as a control for the presence of the puromycin resistance and *EGFP* gene. This knock-in recombinant virus (FV3-GFP) has revealed useful for various studies. FV3-GFP replicate as well as wt-FV3 in BHK cells [44]. Because EGFP expression is under the control of the immediate-early promoter 18K, it should be one of the first viral genes produced upon infection and serves as a good marker of early stages of infection. In cell culture for example, we can detect GFP signal as early as two hours post-infection in a few BHK cells (Figure 2A). GFP fluorescence can be detected in other mammalian cells infected with FV3-GFP when adapted to grow at 30 °C including HeLa and murine macrophage lines like microglial BV2 cells (Figure 2B). In addition, GFP can even be detected in mouse cells infected with FV3-GFP at the non-permissive temperature of 37 °C, which is known to prevent FV3 replication 24 hours post-infection (Figure 2C). This suggests that at the non-permissive temperature FV3 is not only able to bind and infect mammalian cells but is also able to express at least some of its immediate-early genes [45].

More importantly, FV3-GFP can be used to trace infection in vivo. In *Xenopus* *laevis* tadpoles at one day post-infection, GFP signal is detected in the kidney, which is the main site of viral replication. In addition, some GFP signal is also detectable in the brain of tadpoles infected with this recombinant (Figure 2D,E). This observation confirms our recent report that the blood-brain barrier (BBB) is compromised during FV3 infection in tadpole, which results in the dissemination of FV3 in the brain [46].

### 3.2. FV3 KO Mutants.

By analogy to poxviruses and other NCLDV, where more than two dozen viral gene products play critical roles in the evasion of host immunity and virulence, it is postulated that ranaviruses such as FV3 should encode multiple virulence genes. Some putative FV3 virulence or immune evasion genes can be inferred based on homology with genes of eukaryotes or other viruses. However, it is likely that, among the numerous FV3 ORFs that are conserved across ranaviruses but do not share any significant sequence similarity with other viruses or eukaryotes, there are additional, potentially novel, virulence or immune evasion genes. To date four FV3 putative virulence/immune evasion genes have been successfully knocked-out and partially characterized:

1. A viral homolog of the cellular translation factor eIF2α (vIF2α) that is an antagonist of PKR [28]. The vIF2α gene is present and conserved among ranaviruses but is truncated in FV3 and lacks the PKR N-terminal binding and central helicase domains [44].
2. A Caspase Activation and Recruitment Domain (CARD) motif-containing ranavirus gene (vCARD) postulated to interfere with CARD domains containing pro-apoptotic, pro-inflammatory and/or interferon responsive molecules [47,48].
3. A putative ranavirus homolog of β-hydroxysteroid dehydrogenase (vβHSD) that similar to poxviruses may play a role in dampening host immune responses [49,50].
4. An immediate-early gene, 18K, of unknown function but conserved among ranaviruses.

Each FV3 KO mutant was examined in vitro to control for the correct insertion of the puromycin/EGFP cassette at the targeted site and to rule out any contamination by wtFV3. Importantly, compared to wtFV3, all FV3 KO mutants replicate as efficiently in vitro in the mammalian BHK cell line and the fish fat head minnow (FMH) cell line. In contrast, infection of susceptible *Xenopus laevis* (*X. laevis*) tadpoles reveals that the replication and virulence of these FV3 KO mutants is affected in vivo leading to lower levels of mortality than wtFV3. To obtain further insight into the putative virulence/immune evasion mechanisms of these viral genes, the *X. laevis* A6 kidney cell line was used. Unlike the non-amphibian BHK cell line, which does not have a type I interferon response, the replication of 3 FV3 KO mutants (ΔvCARD-, ΔvβHSD- and ΔvIF2α-FV3) were significantly reduced compared to wtFV3 in the *X. laevis* A6 cell line. This is interesting because type I interferon response is elicited by FV3 in A6 cells [51]. Increased sensitivity to amphibian type I IFN was further shown for ΔvCARD- and ΔvIF-2α-FV3 by pretreating A6 cells with *X. laevis* recombinant type I IFN [52], suggesting a role in modulating the cellular interferon response by these FV3 genes.

Further insight into the respective role of these genes in overcoming or subverting host cell defenses by FV3 was obtained by looking at apoptosis induction in A6 cells [52]. Since, interaction of protein with CARD domains is critical in the apoptosis pathway and thus could be interfered by a viral CARD-like gene, it was postulated that disruption of this gene would have an impact on apoptosis during infection. Indeed, ΔvCARD-FV3 triggered significantly more apoptosis than wtFV3 in A6 cells, but surprisingly FV3 with a deletion of the truncated vIF2α (ΔvIF2α-FV3) was also less effective in preventing apoptosis, whereas apoptosis induced by ΔvβHSD-FV3 was comparable to wtFV3. It is noteworthy that vCARD KO mutant induces as much type I IFN as wtFV3, which suggests that vCARD is probably not directly interfering with IFN-I synthesis, but rather may block IFN-induced apoptosis.

The role of the truncated vIF-2α gene in FV3 is enigmatic since it lacks the N-terminal PKR-binding and central helicase domains [44]. These domains have been shown to be critical in vIF2α proteins of ATV [27] and RCV-Z, [28] for counteracting the protein translational arrests mediated by PKR. The impaired replication and virulence of ΔvIF2α-FV3 in in vivo in tadpoles as well as its sensitivity to type I IFN and pro-apoptotic activity, all imply that albeit truncated gene is critical counteracting the programmed cell death induced by viral infection, presumably through the IFN response. It is possible that truncated FV3 vIF2α has adopted unique interactions with PKR, or that FV3 vIF2α may function by targeting a distinct and yet undefined cellular antiviral mechanism.

Although ΔvβHSD-FV3 did also show attenuation of virulence in vivo, which is suggestive of the implication of this gene in promoting FV3 infection, its characterization is in progress.

*18K* is the only gene characterized in FV3 that does not exhibit significant match with other genes outside ranaviruses. Given that it is not an essential gene and that it is expressed at very early stage of infection, it represents an ideal candidate for a novel virulence or immune evasion gene as evidenced by the dramatic growth impairment and reduced lethality in infected tadpole. Notably, FV3-∆18K is more resistant to r*Xl*IFN prestimulation than the three other knockout (KO) mutants, whereas 18K deletion results in substantially increased apoptosis. Similar results were obtained by 18K knockdown with morpholino [41]. These findings suggest a distinctive function of 18K that may be to regulate timely FV3 gene expression and release.

## 4. Other Ranaviruses

In addition to ATV and FV3, other ranaviruses, especially those in Asia and Europe, have been used to generate mutants expressing foreign genes. In fact, one of the first ranaviruses expressing GFP was made in soft-shelled turtle iridovirus (STIV), an Asian ranavirus isolated from infected turtles [43]. This recombinant mutant virus was isolated by selecting for EGFP expressing plaques as the viral envelope protein, VP55, was fused to EGFP. Homologous recombination between a DNA construct containing the VP55-EGFP fusion construct and viral DNA in infected cells generated the knock-in virus, similar to the technique described above for FV3. This recombinant STIV initiated a wave of similar research with other ranavirus isolates from Asian host species.

Rana grylio virus (RGV) has been used to generate a number of knock-out recombinant viruses as well as recombinant virus that can specifically induce expression of the target gene. RGV was isolated from infected pig frogs (*Rana grylio*) and caused high levels of mortality in pig frogs in multiple locations throughout China [53,54]. RGV is genetically similar to FV3 [55] and recombinants deleting non-essential genes have been generated. RGV deleted of the viral envelope protein (ORF 53R) and the thymidine kinase (TK, 92R) was isolated by plaque purification using a plasmid vector recombination cassette that express only GFP [56]. RGV 53R and 92R are not required for growth in cell culture allowing isolation of these mutants without any drug selection. That is, green plaques can easily be observed and recombinant virus purified. Recently, this method of using only a visual marker was used to generate a double deletion RGV expressing EGFP in the TK (92R) locus and red fluorescent protein (RFP) in the deoxyuridine triphosphatase (dUTPase, DUT) (67R) [57]. All of the above RGV mutants were made using plasmid sequences containing DNA to delete the target ORF with a fluorescent marker. These mutants can easily be isolated if the target gene/ORF does not significantly influence viral replication in tissue culture cells. If the gene/ORF is even semi-essential, a single crossover recombination would insert the entire plasmid, including the GFP, without deleting the gene. Although it should be unstable, these events could be propagated in progeny virions with both the target gene and the selection gene being present in the genome. Therefore, confirmation of the site specific deletion is required to ensure the recombinant has not retained the gene while inserting the plasmid, and GFP, into the viral genome. The long-term stability of these mutants, especially during production of high titer viruses remains to be evaluated.

European sheatfish virus (ESV) is the only fish ranavirus used to express foreign genes. In this case, the dihydrofolate reductase gene (DHFR) was knocked-out by homologous recombination using GFP-neomycin resistance [58]. Generation of this mutant was performed using techniques similar to those described above. In this case, the ESV DHFR gene was shown not to play a role in viral replication or pathogenesis in a zebrafish model. This study highlights a fish model system to study ranavirus in vivo as zebrafish have been used to study iridovirus pathogen dynamics [59] and there are many molecular and genetic tools (e.g., transgenic lines with various cell types expressing fluorescent tracers) available that can be used to help characterize ranavirus–host interactions. Characterized ranavirus mutants to date are listed in Table 1.

## 5. Conclusions and Perspective

Although we have now several optimized methods to generate knock-out ranavirus mutants as well as in vitro and in vivo systems to assess the effects of viral gene loss-of-functions, several issues remain that can potentially hamper advancing investigation of these viruses.

A first critical issue is that compared to other DNA viruses, a method to obtain revertants to any KO ranavirus has yet to be developed. This is a critical approach to fully validate that the biological effects observed with knock-out ranavirus mutants are only due to the targeted gene and not by other possible genetic defects. One solution would be to sequence the genome of the recombinant virus to identify any second site mutations [62]. Another difficulty is the selection process to generate recombinants. To introduce a second construct in the genome would require the use of a different selection marker. In our view, a drug selection is important to enforce the stability of the site-specific gene disruption. As summarized above, there have been several reports of gene knock-in in other ranavirus species including RGV that have only used fluorescent reporter gene for selection [56,57,60,61]. Although such a methodology appears to work and may seem simpler, we think that drug selection is safer to minimize the risk of recombination and loss of the insertion during virus production. One possible solution would be to develop a transient dominant selection protocol as used for poxvirus recombinant selection [63]. This would allow for use of drug selection when making the recombinant and could include a fluorescent marker (i.e., GFP or RFP) to visualize the mutant but would not rely on the insertion of a drug selection marker into the virus. Once a recombinant is made and isolated in pure culture, a revertant virus could then be generated by recombination between the mutant virus and a DNA construct containing the gene, and perhaps a tag to identify the revertant, by loss of GFP/RFP during plaque purification.

Another issue concerns the possibility that critical virulence and/or immune evasion may reveal to be essential, and therefore could not be obtained by the existing methodology. Identification of these essential genes can provide valuable insight into their function. In such a case, characterization of these essential genes may require ectopic expression of viral genes or the generation of inducible expression mutants. In the later case, the use of conditional lethal mutants that allow for regulation of expression may help resolve this issue (see below).

As some viral genes likely play roles in both replication and virulence, attempts to knock-out this category of gene can adversely impact the ability to propagate these mutants in culture. Therefore, alternative approaches must be taken to understand gene function. For example, knock-out methodology can be modified to permit analysis of infection using the tetracycline controlled gene expression system [64]. This method permits to propagate virus in vitro (in the presence of the inducer), then use that virus to infect tadpoles (in the absence of inducer) and determine the impact of gene loss in vivo. This system has been used successfully to construct conditional lethal mutants in vaccinia virus and herpesvirus [64,65,66,67,68] and should be adaptable to ranavirus. An alternative method that uses the *E. coli lac* operon as has been successful in generating mutants in VACV and African swine fever virus (ASFV) [69,70,71,72]. Although promising, this technique will need to be further evaluated, as regulation of gene expression is leaky and not 100% efficient.

In comparison to other large DNA viruses such as poxviruses, our understanding of ranavirus gene function is extremely poor. Poxvirus recombination techniques to explore gene function were developed in the 1980s [73,74] and continue to advance today. Therefore, using techniques developed in heterologous systems like those from poxviruses will continue to help advance our progress in understanding the function of ranavirus putative virulence and immune evasion genes. In addition, it may be possible to utilize this well-defined heterologous system to understand ranavirus gene function [36,75]. This approach does have drawbacks, specifically: (i) the higher temperature infection parameters may not resemble the cold-blooded vertebrate host; (ii) requires having an identifiable phenotype; and (iii) orthologous gene swapping. In addition, it is possible that one of the many poxvirus genes may mask ranavirus gene function identification and characterization. Nevertheless, the poxvirus heterologous system is advantageous for multiple reasons: (i) extremely well characterized model viruses in all aspects of the viral life cycle in vitro and in vivo; (ii) easy to manipulate and faster growth rates; (iii) many molecular resources and well defined host–pathogen interactions; and (iv) mechanisms to generate revertant viruses. Therefore, this heterologous system may be useful as a complementary system to study ranaviral gene functions that have broader impacts.

While ranaviruses as expression vectors of foreign genes is relatively recent, significant strides have been made in a relative short time at developing technology that help uncover the function of the large number of genes within this group. In addition, newer powerful technology such as the clustered regularly interspaced short palindromic repeats/Cas9 CRISPR/Cas9 system that has been used to facilitate the generation of knock-out recombinant poxviruses [76,77] are likely applicable to ranaviruses. As we continue to characterize the function of ranavirus genes, we anticipate the continued development of technology, resources and methodology to help in our endeavors to uncover the molecular mysteries of these important pathogens.

## Figures and Tables

**Table 1:** Recombinant Ranaviruses.

Virus	ORF	Predicted Function	Mutant Phenotype	Reporter Marker	Reference
FV3					
	26R	eIF2α homologue	antagonist of PKR; IFN^s^; increased apoptosis; reduced pathogenesis	EGFP-puromycin resistance	[44]
	82R	ICP-18	increased apoptosis; increased induction of type I IFN; reduced pathogenesis	EGFP-puromycin resistance	“
	52L	β-hydroxysteroid dehydrogenase homolog	tbd; reduced pathogenesis	EGFP-puromycin resistance	[52]
	64R	caspase activation & recruitment domain-containing (CARD) protein	IFN^s^; increased apoptosis; reduced pathogenesis	EGFP-puromycin resistance	“
ATV					
	57R	eIF2α homologue	antagonist of PKZ; reduced pathogenesis	neomycin resistance	[27]
	11R	unknown	essential gene	GFP-neomycin resistance	[37]
	25R	RNase III	degrades RNA	GFP-neomycin resistance	“
	40L	CARD-containing gene	tbd; see FV3 above	GFP-neomycin resistance	“
	53R	Unknown—essential	essential gene	GFP-neomycin resistance	“
	54R	unknown	tbd	GFP-neomycin resistance	“
RGV					
	53R	viral envelope protein	green virus	EGFP	[56]
	92R	thymidine kinase (TK)	non-essential	EGFP	“
	53R	viral envelope protein	required for viral production; reduced growth when not expressed	IPTG inducible; EGFP	[60]
	2L	viral envelope protein	required for viral production; reduced growth when not expressed	IPTG inducible; EGFP	[61]
	92R67R	TK and deoxyuridine triphosphatase (dUTPase, DUT)		EGFP/RFP	[57]
ESV					
	114L	dihydrofolate reductase (DHFR)	non-essential	EGFP-neomycin resistance	[58]
STIV					
	VP55	viral envelope protein	green virus	EGFP-VP55 fusion	[43]

tbd = to be determined; IFN^s^ = interferon sensitivity; “ =same reference as above.
